# Proposed Organization of Medical Journal Publishers

**Published:** 1893-09

**Authors:** 


### PROPOSED ORGANIZATION OF MEDICAL JOURNAL PUBLISHERS.

It gives us pleasure to be informed that there is to be organized a Medical Publishers' Association in Washington during the meeting of Pan-American Medical Congress, which convenes Sept. 5.

Such an organization if properly formed will not only be a protection and benefit to the Publishers of Medical Journals, but to the advertisers as well. Whilst the Publishers of many of the Medical Journals are also Editors and probably belong to the Association of Editors which meets simultaneously with the American Medical Association, they certainly must recognize that there are matters pertaining to the business and financial interests of the Journals that do not come within the purview of the Editors' Association. We understand the object of the Publishers' Association shall be for the better protection of legitimate advertisers and the Publishers and is not in any way to take the place of or interfere with the work of the Editors' Association. Such being the case the organization of the Publishers' Association should meet with their hearty co-operation, and we trust that Editors will take note of this proposed movement. Should they themselves not be Publishers of the Journals they edit, we hope they will advise their Publishers of the meeting to be held in Washington Sept. 5, urging them to be present, and give their co-operation to such an organization as shall be for their mutual protection.

